# Examining the anticipated uptake and acceptability of heat therapy in males: Expectations and perceptions before and after hot water immersion

**DOI:** 10.1371/journal.pone.0353797

**Published:** 2026-09-08

**Authors:** David Bellini, Alex Lloyd, Christof A. Leicht, Simon Hodder, Matthew J. Maley

**Affiliations:** 1 Environmental Ergonomics Research Centre, Loughborough University, Loughborough, United Kingdom; 2 School of Sport, Exercise, and Health Sciences, Loughborough University, Loughborough, United Kingdom; 3 The Peter Harrison Centre for Disability Sport, Loughborough University, Loughborough, United Kingdom; University of Hertfordshire, UNITED KINGDOM OF GREAT BRITAIN AND NORTHERN IRELAND

## Abstract

**Background:**

Passive heat therapy (HT), such as hot water immersion (HWI), has emerged as a non-exercise health-promoting intervention. Epidemiological data demonstrate that HT engagement reduces cardiovascular disease risk, however, experimental protocols are often time intensive and perceived as uncomfortable. This study explored the expectations, perceptions and anticipated uptake of HT in response to an acute HWI session.

**Methods:**

Twenty young, healthy, recreationally active males completed a 60-min HWI protocol (39°C) separated by bespoke pre- and post-immersion surveys.

**Results:**

Before immersion, most participants agreed HWI would be easy to complete (90%) and relaxing (85%). Despite reported thermal discomfort during immersion, post-immersion, most participants perceived HWI as enjoyable (60%) and relaxing (65%), although 50% also agreed HWI was more challenging and less enjoyable than expected. Participants indicated that if given autonomy during HWI, the average self-selected duration would have been 31 ± 13 min. Assuming access, the anticipated weekly future engagement with HT was 49 ± 31 min, with 15% of participants indicating they would select HT over walking to improve their health. Time availability emerged as the most influential factor, with all participants agreeing this would impact future engagement.

**Conclusions:**

Although there were positive perceptions of HWI, thermal discomfort and time requirements may limit the uptake of HT as a health-promoting intervention. These findings highlight a discrepancy between the duration of commonly employed HT experimental protocols and what individuals appear realistically willing or able to complete. Future HT research should prioritise acceptability and accessibility by developing time-efficient and thermally tolerable protocols.

## Introduction

Non-communicable diseases (NCDs), including cardiovascular disease (CVD), type 2 diabetes mellitus (T2DM), cancers and chronic respiratory diseases, represent the leading cause of morbidity and mortality worldwide, accounting for 43 million deaths in 2021 [1]. Although NCDs are largely preventable through lifestyle modification, their prevalence continues to rise in part due to an aging population [2]. As such, governments and public health agencies have developed policies prioritising the prevention and management of NCDs through evidence-based interventions [3], although uptake and adherence remain challenging. Whilst regular physical activity and dietary modification are effective primary prevention strategies, only 43% of individuals aged between 16–24 years in England meet physical activity and muscle strengthening guidelines, dropping to 10% in those 75 years and older [4]. Moreover, despite recent breakthroughs in pharmacological treatments of obesity, T2DM and CVD outcomes (e.g., GLP-1 receptor agonists) [5,6], the financial burden associated with NCDs continues to rise, reaching more than $500 billion annually in Europe alone [7]. Furthermore, historical adherence rates to medical interventions often fall below 50% [8,9], partially related to their accompanying unwanted side effects [10]. Although continued research into maximising uptake and adherence of established public health interventions is warranted, development and evaluation of alternative interventions is also of interest through considering their potential benefit to public health as well as adherence rates.

Passive heat therapy (HT) involves the passive elevation of body core temperature, often through modalities such as sauna bathing or hot water immersion (HWI) [11]. Epidemiological data suggests regular HT reduces the risk of all-cause and CVD-related mortality [12,13], resulting in HT receiving attention as a health-promoting intervention. Indeed, there is evidence of beneficial cardiovascular [14–16] and metabolic [11,17] adaptations from chronic HT, such as reduced blood pressure [18] and increased insulin sensitivity [19]. These responses are proposed to be underpinned by the repeated physiological strain experienced during acute heating exposures [20]. Indeed, overlapping physiological effects between HT and exercise [21] have led researchers to describe HT as an “exercise mimetic” for individuals unable or unwilling to exercise [22–27]. However, the translational value of HT is likely to depend not only on physiological efficacy, but also whether it is perceived as sufficiently acceptable, tolerable and practical to support uptake and sustained engagement.

Despite the encouraging beneficial health effects from HT, many protocols remain time-intensive and are often perceived as uncomfortable. For instance, chronic experimental HT interventions commonly involve repeated 60 min exposures [18,19,28–30] and can exceed 150 min per week [18,19,28,30–32], matching the current recommendation for moderate-intensity aerobic physical activity [4]. However, weekly HT engagement between 25 and 80 min has reduced CVD-related mortality relative to reduced engagement groups (≤ 15 min) in epidemiological studies [13], a substantially lower duration than commonly employed experimental protocols.

The mechanisms mediating physiological benefits from HT are in part due to elevations of body core temperature [15], and laboratory HT studies therefore aim to induce such elevations, with increases between 1.0–1.5 °C reported [18,29]. Although direct perceptual comparisons between HT and exercise remain limited, there is some evidence that HWI may elicit more favourable perceptual responses than exercise, whereas dry heat modalities of HT (e.g., sauna or infrared sauna) may exhibit a perceptual profile more similar to exercise [33,34]. Moreover, comparisons between HWI and dry heat exposure have produced mixed findings with respect to favourable perceptual responses [35,36]. Nonetheless, observational evidence suggests that affective responses during HWI may broadly resemble those reported during exercise [29]. More recently, the thermal tolerability of HT has been highlighted [37], and a small number of studies have employed mitigation strategies such as fan use [38], local cooling [39], localised heating protocols [40] and shorter (20 min) heating durations [41]. This is important because perceptions of discomfort and tolerability are likely to influence engagement with HT, particularly if it is to be positioned as a health-promoting intervention. Accordingly, assessing HT perceptions may provide important insight into the barriers and facilitators relevant to adherence and real-world implementation.

Almost all experimental HT research has investigated physiological efficacy rather than real-world translation, where individual preferences, perceived barriers and practical implementation remain largely unexplored. Indeed, there has been limited consideration of systematically examining the future engagement of HT among those who have personally undergone acute heating sessions, drawing on their lived perceptions and the perceived factors contributing to potential uptake. Understanding how laboratory-based HT protocols relate to individual preferences, perceptions and behaviours may help identify barriers and facilitators relevant to uptake and adherence, thereby informing the development of more ecologically valid protocols and improving the relevance of HT as a public health intervention. Therefore, the present study aimed to address this research gap by exploring the expectations, perceptions and anticipated uptake of HT, as well as the factors that may influence engagement. Although the therapeutic application of HT is often targeted towards clinical populations, the present study was designed to examine perceptions rather than physiological efficacy. On this basis, an initial evaluation in a healthy population sample was considered appropriate, enabling investigation of barriers and facilitators to HT engagement without the additional physiological, behavioural and safety-related complexities associated with clinical populations.

## Methods

### Participants

Twenty young, healthy males provided written informed consent and participated in this study between 9 and 25 June 2025 (Table 1). Participants self-reported as recreationally active (≥ 30 min of moderate-intensity exercise at least three times per week for the last three months), non-smoking, working ≥ 35 h per week, either through employment or full-time education as a Doctoral Researcher, and not currently engaging with HT (≥ 4 sessions over the last month). Given the exploratory nature of the study, which aimed to generate initial insight into perceptions, acceptability, and anticipated uptake of HWI rather than to power hypothesis-driven testing of physiological outcomes, no formal a priori power calculation was performed. A sample of 20 participants was considered appropriate for generating preliminary user-informed insight into factors likely to influence engagement with HT. Experimental procedures were approved by the institution’s ethics committee (#21804) and conformed to the Declaration of Helsinki (1964) in all aspects, except registration in a database.

**Table 1:** General characteristics of participants.

	Participants (n = 20)
Age (years)	27 ± 4
Height (cm)	176.7 ± 5.7
Body Mass (kg)	75.6 ± 7.9
BMI (kg/m^2^)	24.2 ± 2.1
Body fat (%)	17.0 ± 3.7
Body surface area (m^2^)	1.92 ± 0.12
IPAQ-S (MET-min/week)	3151 ± 1842

Data are mean ± SD. BMI, body mass index; IPAQ-S, International Physical Activity Questionnaire short version.

### Experimental visit

This was a cross-sectional study involving a single visit where participants completed surveys before and following an acute bout of HWI. Initially, height (Invicta, Bishop, UK), body mass (KCC150, Mettler Toledo, USA) and body fat percentage (MC-780 MA, Tanita, Japan) were measured. Body surface area was calculated using the Du Bois and Du Bois equation [42] and physical activity levels were reported using the International Physical Activity Questionnaire Short Version (IPAQ-S) [43]. Following this, participants were re-informed of the specific HWI protocol that would be completed and then commenced a bespoke 19-item pre-immersion questionnaire assessing their expectations of HWI as well as expected comparisons of HWI to different intensities of physical activity and exercise. See supporting information for complete questionnaire. Once completed, tympanic temperature (T_tymp_) (Thermoscan Pro 6000, Braun, Germany), systolic blood pressure (SBP), diastolic blood pressure (DBP) and heart rate (HR) were measured in duplicate (Tango M2, SunTech Medical, USA), with the mean of the two measurements used for analysis. Thermal comfort (TC) and thermal sensation (TS) were reported using 30 cm visual analogue scales with 30 corresponding to “very comfortable” and “very hot” and 0 reflecting “very uncomfortable” and “very cold”, respectively. Four wireless thermochrons (DS1922L-F50, Maxim Integrated, USA) were then affixed to the left upper triceps, left upper chest, right anterior thigh and right anterior shin using a single piece of adhesive tape (Hypafix, Leukoplast, UK) for calculation of mean skin temperature (T_skin_) [44]. Thereafter, participants entered the water bath (Lay-Z-Spa St. Lucia, Bestway, UK) and submerged to the clavicle while sitting semi-supine (39.0 ± 0.2°C). Following 30 min of immersion, participants sat upright submerged to the umbilicus, were towel-dried above the waist and completed another 30 min. This HWI protocol, which involved a position change from shoulder- to waist-level immersion after 30 min, was utilised to reduce cumulative thermal strain and improve tolerability while maintaining heat exposure [30]. All HWI sessions were conducted under direct researcher supervision, with participants free to terminate the session at any time and removed from the water bath if T_tymp_ exceeded 39°C; no adverse events occurred during experimental testing. Every 15 min throughout immersion, T_tymp_ and T_skin_ were measured, whereas SBP, DBP and HR were re-measured at 30-, 45- and 60-min of HWI, with mean arterial pressure (MAP) calculated as:


$$\begin{array}{c} MAP=DBP+\;\frac{\text{1}}{\text{3}}*(SBP-DBP) \end{array}$$

Immediately following the 60-min immersion, participants exited the water bath, were towel dried, and then completed a 29-item bespoke post-immersion questionnaire during the same experimental visit. The questionnaire assessed post-HWI perceptions, comparisons of HWI to different intensities of physical activity and exercise, anticipated future uptake of HT and perceived barriers and facilitators to engagement; participants were free to leave the laboratory once the questionnaire was concluded.

### Survey data collection

The cross-sectional surveys were designed iteratively and piloted using a small group of both native and non-native English speakers to ensure clarity, comprehension and face validity. Surveys were completed electronically (iPad, Apple, USA) using an online resource (Forms, Microsoft, USA), with a ~ 15 min completion time for each survey. A total of 48 items were constructed to assess the following categories: 1) *Expectations of hot water immersion*; 2) *Perceptions following hot water immersion*; 3) Anticipated f*uture uptake of heat therapy*; 4) *Factors influencing heat therapy adoption*. Questions within the surveys included Likert scales and descriptive formats. The pre- and post-HWI questionnaires are included as supplementary materials.

### Data analysis

Data were analysed on a question-by-question basis. The frequency of survey response data was summarised using descriptive statistics and presented as the percentage of participants selecting a specified response. Likert scale responses were coded using numerical values for determination of the mean and standard deviation. Due to the cross-sectional experimental design of this study, as well as the exploratory nature, one-sample t-tests were utilised to compare the group mean response for each Likert scale item against the neutral midpoint rating (i.e., two on a scale of one to three, and three on a scale of one to five). This approach was employed to determine whether the group mean response significantly deviated from the neutral midpoint, while allowing the interpretation of both the direction and magnitude of the response, and is commonly employed in cross-sectional research involving health and psychology [45–48]. When Likert scale responses were homogeneous, a one-sample binomial sign test was employed. Questions that were repeated pre- and post-immersion were evaluated using paired-sample t-tests and effect sizes reported using Hedges’ g. Thermoregulatory, cardiovascular and perceptual responses throughout HWI were analysed using one-way repeated-measures ANOVA across timepoints (0, 30 and 60 min), with Bonferroni-corrected pairwise comparisons conducted following a significant main effect. ANOVA effect sizes are reported as partial eta squared (ηp^2^) and interpreted as small (0.01–0.059), medium (0.06–0.139) and large (≥ 0.14), whereas pairwise comparison effect sizes are reported as Hedges’ g and interpreted as small (0.2–0.49), moderate (0.5–0.79) and large (≥ 0.80) [49,50]. All statistical analyses were completed using SPSS (v29, IBM, USA) with statistical significance accepted as p < 0.05.

## Results

### Pre-immersion expectations

**Fig 1:**
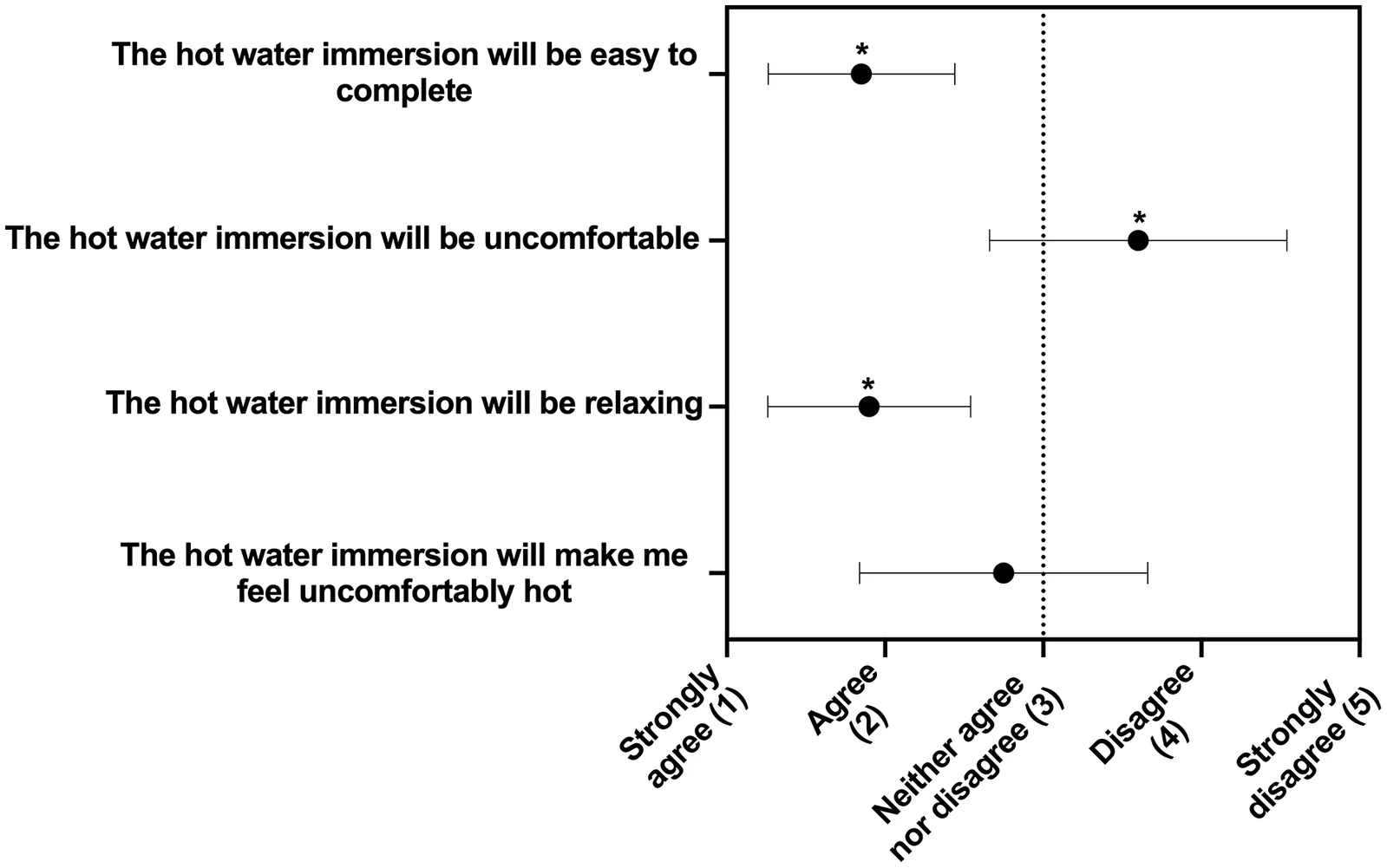
Pre-immersion expectations of hot water immersion. Data are mean ± SD of coded Likert responses and were assessed using a 5-point Likert scale. *Indicates significant difference from neutral midpoint rating (i.e., 3).

Participants’ expectations of HWI that significantly deviated from the neutral midpoint rating (i.e., 3, neither agree nor disagree; Fig 1) included that the HWI would be easy to complete (1.85 ± 0.59; p < 0.001), with 90% of participants selecting “strongly agree” or “agree”. Similarly, expectations that HWI would be uncomfortable (3.60 ± 0.94; p = 0.01), with 60% of participants selecting “strongly disagree” or “disagree”, and that HWI would be relaxing (1.90 ± 0.64; p < 0.001), with 85% selecting “strongly agree” or “agree” also deviated from the neutral rating. Expectations pertaining to being uncomfortably hot during HWI were mixed, where although 40% of participants selected “agree”, 55% selected either “neither agree nor disagree” or “disagree” (2.75 ± 0.91; p = 0.234).

### Hot water immersion exposure

For all thermoregulatory, cardiovascular and perceptual responses, there was a significant main effect of time (p all ≤ 0.006; ηp^2^ = 0.235–0.975) reflecting changes across HWI (Table 2). Bonferroni-corrected pairwise comparisons indicated that all variables were significantly different between 0 and 30 min (p all ≤ 0.001; g = 1.44–9.11) apart from SBP (p = 0.487), and that all variables differed significantly between 0 and 60 min (p all ≤ 0.001; g = 0.91–4.03). Between 30 and 60 min, only T_tymp_, T_skin_, HR, TC and TS changed significantly (p all ≤ 0.001; g = 1.58–4.04), whereas SBP, DBP and MAP were unchanged (p all ≥ 0.283).

**Table 2:** Thermoregulatory, cardiovascular and perceptual responses throughout hot water immersion (HWI).

Parameter	HWI (min)		
	0	30	60
T_tymp_ (°C)	36.8 ± 0.2	38.2 ± 0.4*	37.3 ± 0.3^#^^
T_skin_ (°C)	31.7 ± 0.8	39.0 ± 0.3*	35.8 ± 0.7^#^^
SBP (mmHg)	120 ± 6	118 ± 7	116 ± 6^#^
DBP (mmHg)	72 ± 8	61 ± 8*	61 ± 9^#^
MAP (mmHg)	88 ± 7	80 ± 7*	79 ± 7^#^
HR (bpm)	69 ± 11	104 ± 12*	90 ± 10^#^^
TC (0–30)	25.6 ± 3.9	9.7 ± 4.5*	20.3 ± 5.7^#^^
TS (0–30)	16.4 ± 1.8	24.9 ± 2.7*	19.1 ± 2.9^#^^

Data are mean ± SD. T_tymp_, tympanic temperature; T_skin_, mean skin temperature; SBP, systolic blood pressure; DBP, diastolic blood pressure; MAP, mean arterial pressure; HR, heart rate; TC, thermal comfort; TS, thermal sensation. Significant difference between * 0 and 30 min, ^#^ 0 and 60 min, ^ 30 and 60 min.

### Post-immersion perceptions

**Fig 2:**
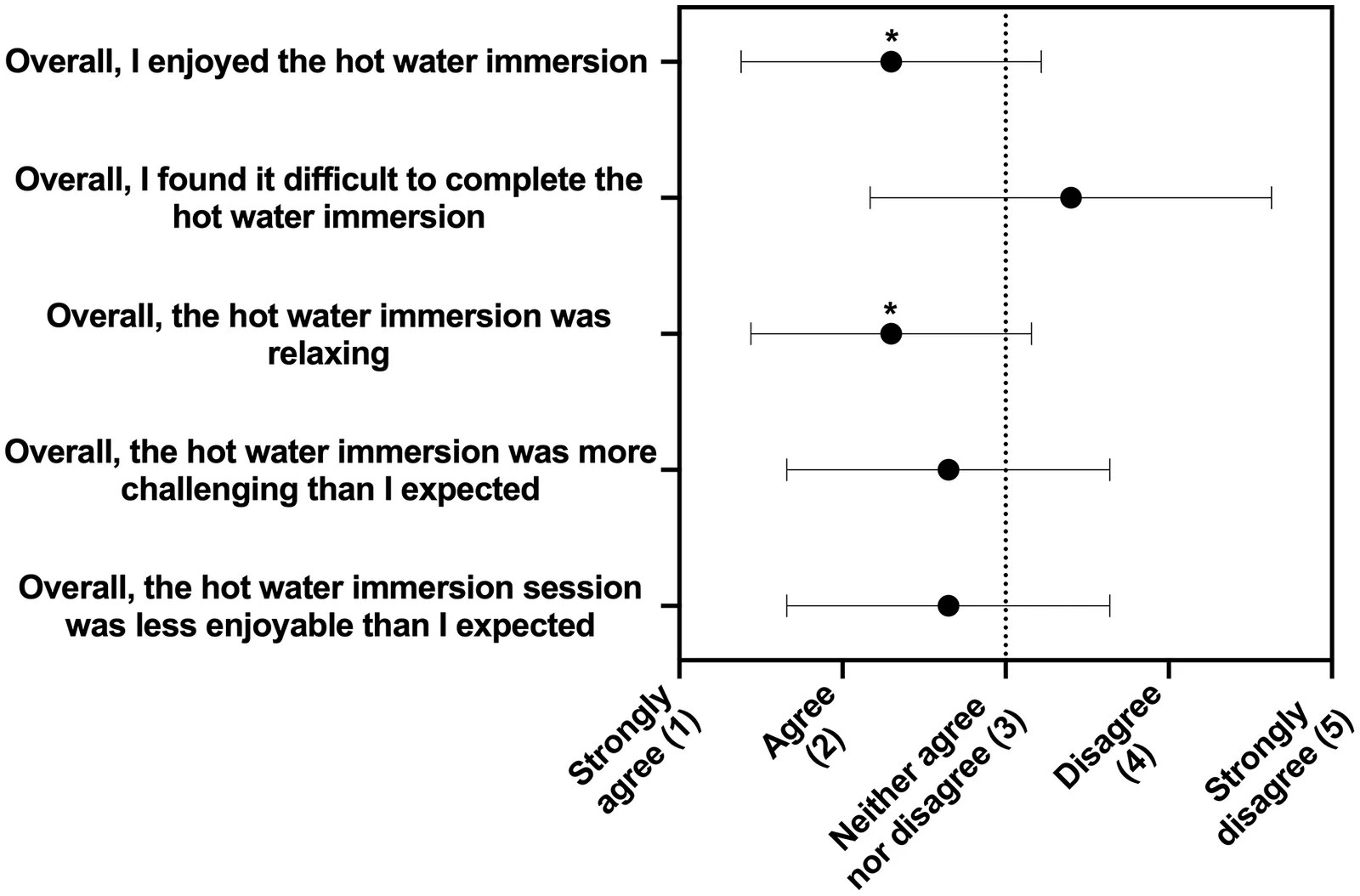
Post-immersion perceptions of hot water immersion. Data are mean ± SD of coded Likert responses and were assessed using a 5-point Likert scale. *Indicates significant difference from neutral midpoint rating (i.e., 3).

Post-HWI perceptions deviating from neutral midpoint (i.e., 3, neither agree nor disagree; Fig 2) included that the HWI session was enjoyable (2.30 ± 0.92; p = 0.003) with 60% of participants selecting “strongly agree” or “agree”, in addition to HWI being perceived as relaxing (2.30 ± 0.86; p = 0.002) where 65% of participants selected “strongly agree” or “agree”. Despite “agree” being the most frequent response with respect to perceiving HWI as being difficult to complete (35%), as well as more challenging (40%) and less enjoyable (45%) than expected, these did not significantly deviate from neutral (p ≥ 0.130). In terms of quantifying preferences for a hypothetical self-selection of HWI duration, participants reported they would have completed a total of 31 ± 13 min, consisting of 20 ± 7 min of shoulder-level immersion followed by 11 ± 10 min of waist-level immersion if the protocol was not prescribed and they had behavioural autonomy.

### Comparisons with physical activity and exercise

**Fig 3:**
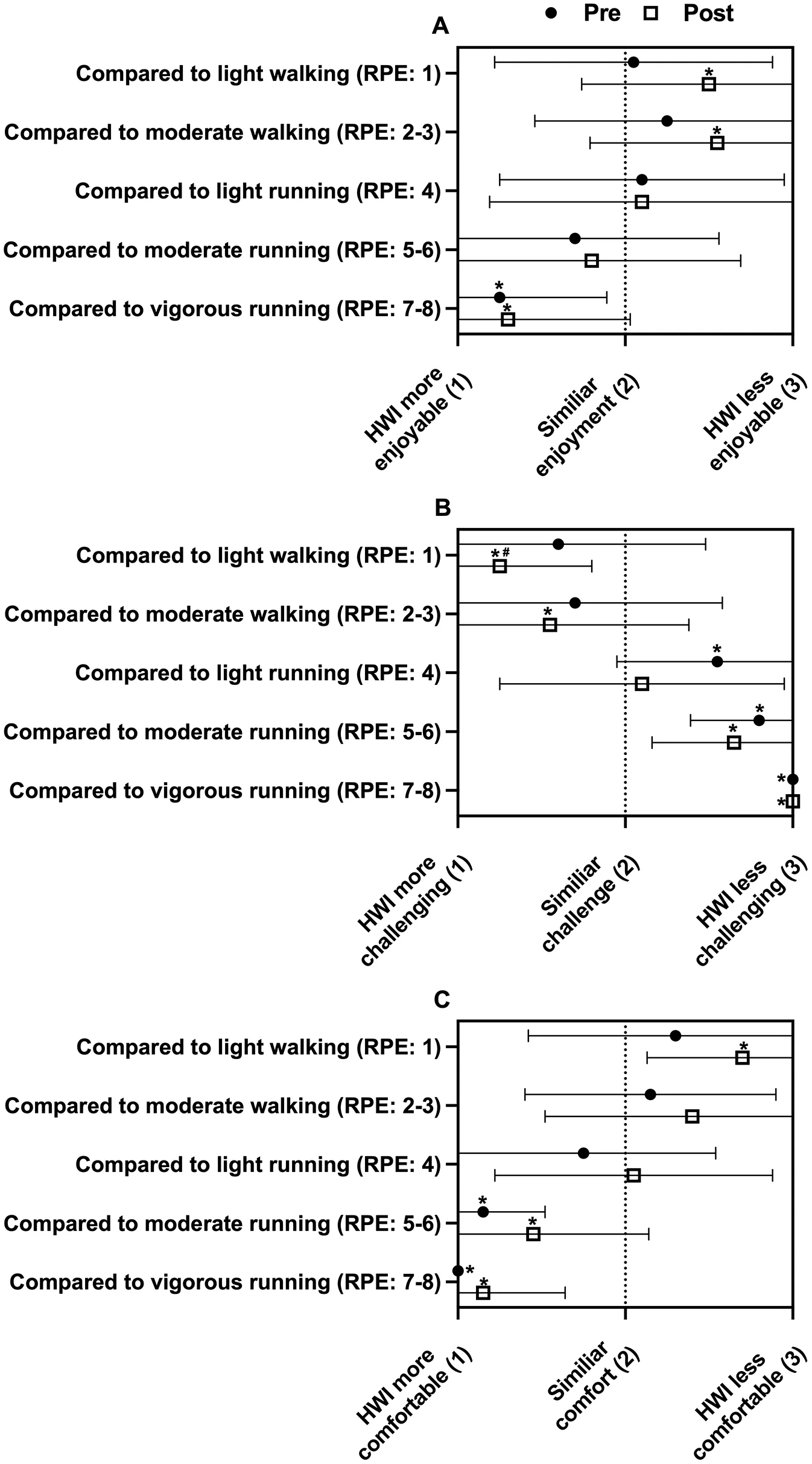
Pre (circles) and Post (squares) hot water immersion (HWI) expectations of enjoyment (A), challenge (B) and comfort (C) when compared to physical activity and exercise. RPE, rating of perceived exertion. Data are mean ± SD of coded Likert responses and were assessed using a 3-point Likert scale. *Indicates significant difference from neutral midpoint rating (i.e., 2). ^#^indicates significant difference between pre- and post-immersion responses.

When participants were asked at pre- and post-immersion to compare HWI with various intensities of physical activity and exercise, pre-immersion expectations of enjoyment during HWI did not significantly deviate from the neutral midpoint response (i.e., 2, similar levels of enjoyment), with the exception of greater enjoyment during HWI compared with vigorous running (1.25 ± 0.64; p < 0.001; Fig 3A). Post-immersion, enjoyment during HWI also deviated to a similar degree from vigorous running (1.30 ± 0.73; p < 0.001), but was rated as less enjoyable compared with light (2.50 ± 0.76; p = 0.008) and moderate walking (2.55 ± 0.76; p = 0.004). However, there were no significant differences between pre- and post-immersion responses for any activity comparison (p ≥ 0.058).

With respect to challenge comparisons, pre-immersion expectations of HWI deviated from the neutral response (i.e., 2, similar levels of challenge) for each intensity of running (p < 0.001; Fig 3B). Post-immersion, comparisons deviated from similar levels of challenge for all activities apart from light running (p ≤ 0.025). Furthermore, post-immersion responses significantly moved towards HWI being perceived as more challenging when compared with light walking relative to pre-immersion (p = 0.049; g = 0.451), whereas other activity comparisons were not significantly different (p ≥ 0.095).

Lastly, pre-immersion comfort expectations of HWI deviated from the neutral response (i.e., 2, similar levels of comfort) for moderate and vigorous running (p < 0.001; Fig 3C). Post-immersion, comfort comparisons to moderate and vigorous running also deviated from similar levels, in addition to light walking (p ≤ 0.002). However, there were no significant differences between pre- and post-immersion responses for any activity comparison (p ≥ 0.055).

### Heat therapy uptake and considerations

**Fig 4:**
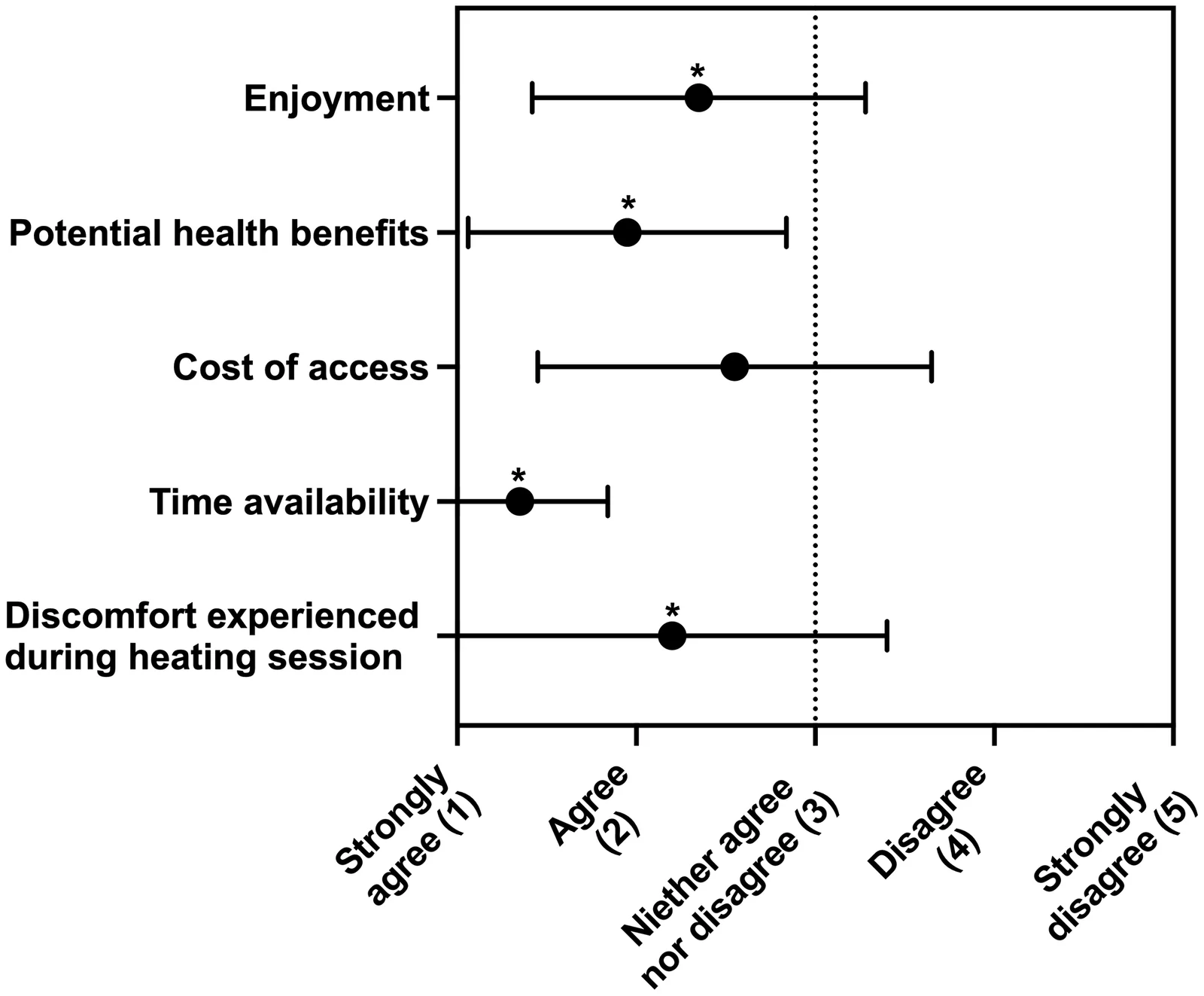
Post-immersion perceptions of facilitators and barriers of future anticipated uptake of heat therapy. Data are mean ± SD of coded Likert responses and were assessed using a 5-point Likert scale. *Indicates significant difference from neutral midpoint rating (i.e., 3).

When asked about anticipated future HT uptake assuming access to their preferred method, participants reported an average of 49 min of HT per week (Table 3). However, only 15% of participants indicated that with access they would select HT over walking to improve their health. Lastly, when asked to indicate which factors would influence the uptake of HT going forward (Fig 4), all responses apart from “cost of access” significantly deviated (p ≤ 0.008) from neutral midpoint (i.e., 2, neither agree nor disagree), with “time availability” displaying the highest agreement with 100% of participants selecting either “strongly agree” or “agree”.

**Table 3:** Anticipated uptake of heat therapy post-immersion.

Variable	Subcategory	Results		
		Mean	SD	Range
**Future uptake of heat therapy**	Sessions per week	1.7	1.2	0-5
	Duration per session (min)	29	14	0-60
	Total weekly time (min)	49	31	0-125
		**Percentage of participants**		
**Preference for improving health going forward**	Heat therapy	15%		
	Walking	85%		

## Discussion

This study aimed to explore the expectations, perceptions and anticipated uptake of HT in individuals before and after a single HWI exposure, thereby evaluating perspectives among those familiar with the realistic demands of HWI. The findings demonstrate that, although pre-immersion expectations of HWI were positive and HWI was regarded as relaxing and enjoyable post-immersion, the thermal discomfort experienced during the exposure was highlighted as challenging. Consequently, participants indicated they would have completed HWI for roughly half of the prescribed duration if given autonomy (i.e., ~ 31 total min). Anticipated future engagement of HT (~49 min per week) was substantially less than what is commonly employed in previous experimental protocols [18,19,28,30,31], where participants indicated time availability as the primary barrier to uptake. Despite epidemiological evidence that relatively modest engagement with HT can yield health benefits [13], our findings suggest a disparity between the duration of frequently employed experimental HT protocols and what individuals appear realistically willing or able to complete.

### Perceptions of hot water immersion

Pre-immersion expectations of HWI were generally optimistic, where the majority of participants anticipated the exposure would be easy to complete and relaxing, and disagreed that it would be uncomfortable. These perceptions align with individuals regularly engaging with HT, where relaxation (100%) and enjoyment (99.6%) were highlighted as motivations of engagement within active sauna users [51]. Following HWI, perceptions remained positive for certain aspects, including that the majority of participants regarded HWI as enjoyable and relaxing, though they also agreed that the exposure was more challenging and less enjoyable than expected. Furthermore, direct pre- to post-immersion comparisons showed only limited changes in how HWI was perceived relative to physical activity and exercise, with the only significant shift being that HWI was perceived as more challenging when compared with light walking. Nevertheless, following immersion, HWI was generally perceived less favourably than walking, broadly similar to light running, and in some perceptual domains more favourably than moderate-to-vigorous running, suggesting that, despite the passive aspect of HWI, there may be comparable perceptual responses to physical activity and exercise.

Participants indicated that if they had behavioural autonomy during HWI, they would have immersed themselves on average for 31 min. When considering real-world engagement data, e.g., Sauna use in the general population, heat exposures are often less than the 60-min duration used in this study, typically ranging between 15 and 50 min with intermittent breaks [12,13,51]. However, it is noteworthy that our participants’ anticipated future engagement with HT aligns with real-world engagement data. For instance, Hussain and colleagues [51] described that in 460 active sauna users the median sauna sessions per month was six with the total exposure time being 49 min per sauna, extrapolating to ~75 min per week. Moreover, in 998 Finnish adults aged 18–75 the median sauna bathing frequency was “two to three times a week” [52], whereas among 271 northern Swedish sauna bathers the median exposure duration session was 15–20 min per sauna session split between one or two bouts [53]. Our data are also in line with epidemiological evidence often cited to underpin HT research, where 1688 Finnish men and women reported a median total duration of 30 min of sauna bathing per week [13]. Importantly, within epidemiological studies weekly sauna engagement between 25 and 80 total min per week has resulted in reductions in all-cause and CVD-related mortality [12,13], further highlighting the gap between currently employed HT interventions and real-world engagement.

### Future uptake of heat therapy

Although the HWI exposure was deemed enjoyable and relaxing, participants reported that, assuming access, they would expect their future engagement with HT to be an average of 49 min per week. This is substantially lower than typically prescribed experimental HT interventions in which chronic protocols commonly exceed 150 min per week [18,19,28,30–32]. Similarly, the aerobic aspect of current physical activity guidelines suggests 150 min of activity per week, however, importantly, over one third of adults currently do not achieve this [4]. Given that chronic HT interventions commonly involve exposure durations of an hour or more multiple times a week [18,19,28–30,32], greater single session durations than the entire anticipated weekly uptake reported in this study, it seems unlikely from a time commitment perspective that the HT protocols commonly employed within laboratories can effectively translate to real-world adoption.

The anticipated future uptake of HT should be considered in the context of the acute physiological and perceptual strain imposed by the HWI protocol. In the present study, thermoregulatory, cardiovascular and perceptual strain increased during the first 30 min of HWI, with T_tymp_, T_skin_, HR, TC and TS then partially alleviated following the transition to waist-level immersion, whereas SBP, DBP and MAP remained stable between 30 and 60 min. This pattern is relevant because the beneficial adaptations elicited by HT are thought to be underpinned by the repeated physiological strain experienced during acute heat exposure [20]. Although T_tymp_ was measured in this study, previous research from our group using the same HWI protocol has demonstrated a ~ 1.0°C increase in rectal temperature [54], suggesting that the more modest T_tymp_ response (~0.6°C) may underestimate the rise in body core temperature. Notably, this thermoregulatory strain is considerably lower than that observed in many chronic HT interventions, which can exceed a rectal temperature increase of 1.5°C [18,19,28,29], as the protocol employed in this study was purposefully designed to be less thermally challenging to improve tolerability. Despite this relatively lower thermoregulatory burden, the duration and discomfort experienced during HWI were nonetheless perceived as barriers, further emphasising that current experimental HT protocols require significant modification to address user-reported engagement barriers. These findings align with broader public health intervention adherence, where perceptions of effort or discomfort often hinder long-term adherence [55,56].

Adherence to medical and health interventions is critical for effective healthcare and preventative medicine, yet uptake remains persistently low. Even for simple interventions such as oral medications, real‑world adherence rates often fall below 50%, with systematic reviews reporting that 30–50% of patients with chronic diseases fail to follow prescribed regimens [8,9]. Self‑reported full adherence to pharmacotherapies for conditions such as hypertension, diabetes, hyperlipidemia, and cardiovascular disease can be as low as 25%, with at least one‑third of patients repeatedly failing to follow medication recommendations [9]. If adherence is poor for low‑burden interventions such as oral medication, it is reasonable to expect even greater limitations for more effortful and time‑consuming interventions such as diet, exercise, and HT, which demand sustained behavioural change and are undermined by the same factors that constrain pharmacotherapy adherence – side effects, limited health literacy, functional decline, socioeconomic constraints, psychosocial barriers, fragmented care pathways, and poor provider-patient communication [8,9,57,58]. Without acknowledging these determinants, lifestyle interventions such as HT risk remaining theoretical ideals rather than practical, sustainable solutions for the very populations they are intended to serve.

### Practical considerations of implementation

Translating HT from laboratories to a scalable public intervention presents several logistical and behavioural challenges. We provide evidence for this, as only 15% of participants indicated that given access, they would choose any form of HT over walking to improve their health. Furthermore, when participants were asked about potential factors impacting their future uptake of HT, time availability appeared to be the most influential factor, with 100% agreeing that it would influence their engagement. Perceived discomfort and time demands are not unique to HT and have been previously highlighted as barriers to exercise engagement [55,59,60]. However, as HT is generally recommended preventatively for long-term risk reduction [11], greater attention should be placed on maximising the acceptability rather than the physiological efficacy. Furthermore, as with any public health intervention, it is important to account for the total time required beyond the activity itself. For example, a HT exposure lasting 30 min may realistically require more than twice the time commitment when considering the associated preparation and cool down (e.g., travel, showering, cooling down, changing clothes). Ultimately, sustained engagement will underpin the successful implementation of HT, analogous to distinctions between structured exercise prescription and general physical activity promotion [59–61].

Another potential barrier to the widespread adoption of HT relates to access, impacted by both socioeconomic and cultural factors. For instance, Finland is frequently cited as a model for HT engagement due to high rates of sauna use, where approximately 60% of adults report using a sauna at least once a week [52]. However, this is in part facilitated by domestic availability as these authors suggest new houses built in Finland have increasingly come with saunas since the mid 20^th^ century, highlighting the requirement to address the access barrier for broad HT adoption. Indeed, whether similar HT adoption will extend to countries without such infrastructure and cultural embeddedness warrants consideration.

Safety is an additional, though almost entirely overlooked, area of concern surrounding the broader implementation of HT. In Japan, forensic analysis of unintended mortality rates suggests somewhere between 6,000 [62] and 19,000 [63] deaths per year are bathtub-related, with the majority of sudden deaths in older adults due to ischemic heart disease [64]. Conversely, there is epidemiological data reporting an inverse relationship between bathing frequency and CVD incidence in middle-aged Japanese adults [65]. Nonetheless, older adults exhibit heightened susceptibility to thermoregulatory and cardiovascular strain during passive heat stress as a result of diminished function [66,67]. This may be particularly concerning given older adults display reduced perceptions of thermal sensitivity to heat [68], potentially reducing behaviour adjustments during heating and consequently increasing thermal strain [69], highlighting the requirement to account for population-specific risks. Accordingly, implementing HT in vulnerable populations should be approached cautiously and supported by appropriate screening, supervision and protocol modification. Potential safety strategies may include limiting heat strain through lower initial temperatures, shorter exposure durations, reduced immersion depths, gradual progression across repeated sessions and, where appropriate, medical oversight.

### Recommendations

Several recommendations emerge for researchers and public health professionals to enhance the viability of HT as an effective public health intervention. First, future research should identify the minimum effective dose of HT capable of eliciting meaningful health benefits, as advocated for previously [70], as currently employed laboratory protocols appear substantially more time-intensive than real-world engagement patterns [37]. Second, the thermal tolerability of HT should be addressed, including through mitigation strategies such as fan use [38] and local cooling [39,71], or by allowing more flexible, behaviour-based adjustments to heating durations or exposure areas rather than prescribing rigidly controlled protocols. Third, HT may warrant consideration beyond long-term disease prevention, where positive outcomes for specific use-cases have been described for muscle rehabilitation [72], exercise performance [73] and to augment the effects of exercise [74], sleep quality [75], mental health disorders [76], seasonal inactivity during winter months [77] and symptom management [78]. Finally, physiology researchers may benefit from employing mixed-methods approaches for a more holistic evaluation of HT. For example, integrating psychology and behaviour change science could result in a better understanding of how perceptions associated with physiological strain translate to real-world adoption. Collectively, these principles may help HT research transition from an efficacy-centric to a more engagement-centric focus, balancing measurable health outcomes with real-world feasibility and adherence.

### Limitations and considerations

The bespoke questionnaires were developed specifically for the present research, as no validated questionnaire was available to assess perceptions, anticipated uptake and acceptability of HWI. These questionnaires were not psychometrically validated beyond face validity and should therefore be interpreted as exploratory, context-specific tools rather than definitive or generalisable measures of HT acceptability. Furthermore, participants were not formally familiarised with walking and running comparators, and responses may therefore have been influenced by preconceived perceptions of activities such as vigorous running. Additionally, while rectal temperature is typically used to reflect body core temperature in HT studies evaluating physiological efficacy, T_tymp_ was utilised as a less invasive index of body core temperature to minimise any potential effects on perceptual responses. However, T_tymp_ is comparatively lower than rectal temperature and can be influenced by ear canal anatomy and environmental conditions [79], which should be considered when interpreting the relatively modest thermoregulatory responses observed.

A cross-sectional design was utilised to explore perceptions of HT following a single HWI exposure. Consequently, first-impression responses were captured regarding future engagement rather than long-term adherence, and stated intentions may not reflect actual health-related behaviours [80,81]. Nonetheless, the observed changes in perception before and after HWI may reflect the replacement of preconceived expectations with direct experience of the intervention, suggesting that perceptions of HWI may not fully capture the practical realities of the exposure. Although broader questionnaire-based responses were assessed before and after HWI to capture expectations and perceptions of the overall exposure, perceptual monitoring during immersion was limited to TC and TS, meaning richer subjective data, such as open-ended responses, were not captured. Participants currently engaging with HT were not recruited, as the study aimed to characterise perceptions following an initial HWI exposure rather than responses influenced by prior HT engagement, thereby better capturing barriers and facilitators to initial engagement. Accordingly, it remains unknown whether these perceptions would persist or change following long-term habituation, an important consideration as chronic HT can elicit beneficial physiological and perceptual adaptations which may help facilitate sustained engagement [18,29]. In addition, as experimental testing was conducted during June and local environmental heat exposure was not formally assessed, seasonal heat acclimatisation may have influenced responses to some extent.

The participant sample consisted only of young, healthy, recreationally active males, restricting the generalisability of our findings. Females were not included as sex differences in thermoregulatory and perceptual responses have been reported during passive heating [71,82], which would have introduced additional variability beyond the scope of this exploratory study. Furthermore, in populations where HT may be most beneficial, including older adults, overweight or obese individuals and clinical groups, reduced thermoregulatory capacity compared with young healthy individuals [66,83,84] may present additional implementation challenges, particularly from a safety perspective. Collectively, these considerations suggest that the perceptions observed in our young, healthy male sample may be more favourable than those likely to be observed in populations with greater thermoregulatory or perceptual challenges; accordingly, future work should investigate populations more representative of the general public. Additionally, perceptions were assessed in a controlled research environment, which contrasts with the wellness-oriented settings in which HT is often practiced, where engagement may also be influenced by factors such as social context, accessibility, cultural norms and competing time demands. Although the present healthy, non-sedentary sample may report HWI and exercise as acceptable or enjoyable, this is unlikely to be experienced to the same extent in sedentary or clinical populations, the groups at whom HT is primarily targeted. More broadly, HT may preferentially appeal to individuals who already meet physical activity guidelines and are generally health conscious. For example, active sauna users have been reported to be predominantly non-smokers and regularly exercising individuals [51], while epidemiological evidence suggests limited additional benefit of more frequent sauna exposure in men with high cardiorespiratory fitness [85]. Together, these findings suggest that habitual sauna bathing may cluster with other favourable health behaviours rather than preferentially attracting sedentary populations. This potential “healthy user” pattern is important because HT is often framed as an alternative or adjunct for those unwilling or unable to exercise; however, if uptake is greatest among already-active individuals, the public health impact may be substantially smaller than anticipated.

## Conclusions

This study provides an examination of the perceptions and anticipated uptake of HT in response to a single 60-min HWI exposure. While HWI was perceived as relaxing and enjoyable, time availability appeared as the most practical barrier influencing future adoption, followed by thermal discomfort. The substantial disparity between expected future uptake of HT, which aligns with real-world engagement data, and HT protocols currently employed in experimental interventions highlights an important opportunity to bridge the gap between physiological efficacy and real-world implementation. To enhance the viability of HT as a public health intervention, future research should focus on developing time-efficient and thermally tolerable HT protocols, promoting acceptable, accessible and safely implementable real-world adoption.

## Supporting information

S1 Data Thermoregulatory, cardiovascular, perceptual and questionnaire data included in this study. (XLSX)

S2 Questionnaires Pre and post questionnaires. (DOCX)
