# Supplementary material for: Examining the anticipated uptake and acceptability of heat therapy in males: Expectations and perceptions before and after hot water immersion
Source: PLoS One. 2026 Sep 8;21(9):e0353797. doi: 10.1371/journal.pone.0353797 (PMC13552788; doi:10.1371/journal.pone.0353797)
Supplement: Supplementary file 1 [file pone.0353797.s002.docx]

# Pre Immersion Questionnaire

## Background Information

Before starting the questionnaire, please read through the below background information on Heat Therapy. After you have read this information, please check the box below and proceed to the questionnaire.

### 1. Heat therapy is an intervention which involves the passive exposure to external heat sources, raising your body’s core temperature. Common methods of heat therapy include saunas, steam rooms and hot water immersion (for example taking a hot bath). This questionnaire will explore your expectations of the hot water immersion exposure.

I have fully read and understood the above information

## Hot Water Immersion Expectations

You will complete 60 minutes of hot water immersion at 39°C where the first 30 minutes the water will be at shoulder level and the next 30 minutes the water at waist level.

### 2. Please indicate below how you feel about the following statements related to the hot water immersion protocol

| Strongly agree | Agree | Neither agree nor disagree | Disagree | Strongly disagree |
| --- | --- | --- | --- | --- |

The hot water immersion will be easy to complete

The hot water immersion will be uncomfortable

The hot water immersion will be relaxing

The hot water immersion will make me feel uncomfortably hot

### 3. Please indicate below how enjoyable you think the hot water immersion will be to complete compared to different intensities of physical activity and exercise for the same total duration (60 minutes).

Compare activities using the rating of perceived exertion (PRE) scale provided

| Hot water immersion more enjoyable | Similar enjoyment | Hot water immersion less enjoyable |
| --- | --- | --- |

Compared to light walking (RPE: 1)

Compared to moderate walki ng (RPE: 2-3)

Compared to light running (R PE: 4)

Compared to moderate running (RPE: 5-

6)

Compared to vigorous running (RPE: 7-

8)

### 4. Please indicate below how challenging you think the hot water immersion will be to complete compared to different intensities of physical activity and exercise for the same total duration (60 minutes).

Compare activities using the rating of perceived exertion (PRE) scale provided

| Hot water immersion more challenging | Similar Challenge | Hot water immersion less challenging |
| --- | --- | --- |

Compared to light walking (RPE: 1)

Compared to moderate walki ng (RPE: 2-3)

Compared to light

running (RPE: 4)

Compared to moderate running (RPE: 5-

6)

Compared to vigorous running (RPE: 7-

8)

### 5. Please indicate below how comfortable you think the hot water immersion will be to complete compared to different intensities of physical activity and exercise for the same total duration (60 minutes).

Compare activities using the rating of perceived exertion (PRE) scale provided

| Hot water immersion more comfortable | Similar comfort | Hot water immersion less comfortable |
| --- | --- | --- |

Compared to light walking (RPE: 1)

Compared to moderate walki ng (RPE: 2-3)

Compared to light

running (RPE: 4)

Compared to moderate running (RPE: 5-

6)

Compared to vigorous running (RPE: 7-

8)

# Post Immersion Questionnaire

## Post hot water immersion perceptions

1. Please read the questions below and select an answer most appropriate for you in relation to the hot water immersion session overall

| Strongly agree | Agree | Neither agree nor disagree | Disagree | Strongly disagree |
| --- | --- | --- | --- | --- |

Overall, I enjoyed the hot water immersion

Overall, I found it difficult to complete the hot water immersion

Overall, the hot water immersion was relaxing

Overall, the hot water immersion was more challenging than I expected

Overall, the hot water immersion was less enjoyable than I expected

### 2. Please indicate below how enjoyable the hot water immersion was compared to different intensities of physical activity and exercise for the same total duration (60 minutes)

Compare activities using rating of perceived exertion scale (1-10) provided

| Hot water immersion more enjoyable | Similar enjoyment | Hot water immersion less enjoyable |
| --- | --- | --- |

Compared to light walking (RPE: 1)

Compared to moderate walking (RPE: 2-3)

Compared to light running (R PE: 4)

Compared to moderate running (RPE: 5-

6)

Compared to vigorous running (RPE: 7-

8)

### 3. Please indicate below how challenging the hot water immersion was compared to different intensities of physical activity and exercise for the same total duration (60 minutes).

Compare activities using rating of perceived exertion scale (1-10) provided

| Hot water immersion more challenging | Similar challenge | Hot water immersion less challenging |
| --- | --- | --- |

Compared to light walking (RPE: 1)

Compared to moderate walking (RPE: 2-3)

Compared to light running (R PE: 4)

Compared to moderate running (RPE: 5-6)

Compared to vigorous running (RPE: 7-8)

### 4. Please indicate below how comfortable the hot water immersion was compared to different intensities of physical activity and exercise for the same total duration (60 minutes).

Compare activities using rating of perceived exertion scale (1-10) provided

| Hot water immersion more comfortable | Similar comfort | Hot water immersion less comfortable |
| --- | --- | --- |

Compared to light walking (RPE: 1)

Compared to moderate walking (RPE: 2-3)

Compared to light running (R PE: 4)

Compared to moderate running (RPE: 5-6)

Compared to vigorous running (RPE: 7-8)

### 5. Please outline the specific hot water immersion protocol you would have completed had it not been prescribed

For example: shoulder-level x minutes, waist-level y minutes

## Future Uptake of Heat Therapy

### 6. Please indicate how many times over the course of a week you would be likely to complete heat therapy if you had access to any of the above methods

0

1

2

3

4

5

6

7

### 7. Please indicate how many minutes for each heat therapy session per week you would be likely to complete going forward if you had access

### 8. Please indicate which factors would influence your decision to complete heat therapy going forward

| Strongly agree | Agree | Neither agree nor disagree | Disagree | Strongly Disagree |
| --- | --- | --- | --- | --- |

Time availability Cost of access Enjoyment

Potential health benefits

Discomfort experienced during heating session

### 9. Going forward to improve your health with access to any method of heat therapy, would you prefer heat therapy or walking of the same duration?

Walking

Heat therapy
